# The MOBILE Study—A Phase IIa Enriched Enrollment Randomized Withdrawal Trial to Assess the Analgesic Efficacy and Safety of ASP8477, a Fatty Acid Amide Hydrolase Inhibitor, in Patients with Peripheral Neuropathic Pain

**DOI:** 10.1093/pm/pnx046

**Published:** 2017-04-05

**Authors:** Daniel Bradford, Anjali Stirling, Etienne Ernault, Maggie Liosatos, Katherine Tracy, Jennifer Moseley, Paul Blahunka, Mike D Smith

**Affiliations:** 1 Astellas Pharma Europe B.V., Leiden, the Netherlands; 2 Astellas Pharma Global Development, Northbrook, Illinois, USA

**Keywords:** Peripheral Neuropathic Pain, Fatty Acid Amide Hydrolase Inhibitor, ASP8477, Enriched Enrollment Randomized Withdrawal, Painful Diabetic Peripheral Neuropathy, Postherpetic Neuralgia

## Abstract

**Objective:**

To evaluate the analgesic efficacy and safety of ASP8477 in patients with peripheral neuropathic pain (PNP).

**Design:**

Enriched enrollment randomized withdrawal.

**Setting:**

Centers in Poland (four), Czech Republic (six), and the United Kingdom (two).

**Subjects:**

Patients aged 18 years or older with PNP resulting from painful diabetic peripheral neuropathy or postherpetic neuralgia.

**Methods:**

A four-week screening period followed by a single-blind period (six-day dose titration and three-week maintenance period with ASP8477 [20/30 mg BID]). Treatment responders (defined as a ≥30% decrease in the mean average daily pain intensity during the last three days of the single-blind period) were stratified by disease and randomized to receive placebo or continue ASP8477 during a three-week, double-blind, randomized withdrawal period. The primary end point was change in mean 24-hour average numeric pain rating scale (NPRS) from baseline to end of double-blind period.

**Results:**

Among 132 patients who enrolled, 116 entered the single-blind period and 63 (ASP8477, N = 31; placebo, N = 32) completed the double-blind period. There was no difference in mean 24-hour average NPRS score (*P *=* *0.644) or in time-to-treatment failure (*P *=* *0.485) between ASP8477 and placebo. During the single-blind period, 57.8% of patients were treatment responders. ASP8477 was well tolerated. During the single-blind period, 22% of patients experienced at least one treatment-related adverse event (TEAE); during the double-blind period, 8% in the ASP8477 arm and 18% in the placebo arm experienced at least one TEAE.

**Conclusions:**

ASP8477 was well tolerated in patients with PNP; however, ASP8477 did not demonstrate a significant treatment difference compared with placebo.

## Introduction

Peripheral neuropathic pain (PNP), a complex chronic syndrome resulting from lesions to the peripheral nervous system, is caused by multiple etiologic factors (i.e., mechanical trauma, metabolic diseases, infections, or tumor invasion) [1,2]. Common causes of PNP are diabetic neuropathy and postherpetic neuralgia (PHN) [3]; however, because these are complex conditions, eliminating the underlying etiology can be challenging. Consequently, the use of targeted therapies to manage discomfort, or to reduce the sensory hypersensitivity usually observed following the onset of neuropathic pain, is important [4]. There are a number of approved treatment options for neuropathic pain including pregabalin and gabapentin (which act via the neurotransmitter gamma-aminobutyric acid [5–10]) and duloxetine (a serotonin norepinephrine reuptake inhibitor [6,9,11–13]). Nevertheless, there is an unmet need for new treatments with novel mechanisms of action that can further reduce pain in patients with PNP.

The enzyme fatty acid amide hydrolase (FAAH) is responsible for the degradation of several endogenous fatty acid amides, including the endocannabinoids anandamide (AEA), oleoylethanolamide (OEA), and palmitoylethanolamide (PEA) [14]. Endocannabinoids participate in a variety of biological activities including those related to pain. The effects of endocannabinoids are mediated by their binding to cannabinoid receptors, the activation of which is known to produce analgesic effects [15]. Inhibiting FAAH enzymes has been shown to increase the level of AEA, OEA, and PEA; thus, FAAH inhibitors may be beneficial in the management of neuropathic pain. This analgesic effect has been demonstrated in several preclinical studies [15–17].

ASP8477, a novel, potent, selective inhibitor of FAAH, selectively inhibits human FAAH-1 activity in vitro with a 50% inhibitory concentration (IC_50_) value of 3.99 (unpublished data). In phase I studies, ASP8477 was shown to increase plasma endocannabinoid concentrations in healthy subjects (unpublished data). Multiple doses of ASP8477 of 60 mg or less daily were also well tolerated and exhibited analgesic properties in an integrated pain model in healthy volunteers (unpublished data).

Here, we report the findings of a phase IIa enriched enrollment randomized withdrawal (EERW) trial designed to assess the analgesic efficacy, safety, and tolerability of ASP8477 in patients with PNP resulting from painful diabetic peripheral neuropathy (PDPN) or PHN.

## Methods

### Study Overview

The phase IIa EERW MOBILE study was designed to assess the analgesic efficacy and safety of ASP8477 in patients with PNP resulting from PDPN or PHN (NCT02065349). The study was a multinational, multicenter study conducted at 12 centers in three countries: Poland (four centers), the Czech Republic (six centers), and the United Kingdom (two centers). Prior to the study, independent ethics committee (IEC) approvals were obtained for ethical, scientific, and medical appropriateness of the study and IEC-approved written informed consent was obtained from each patient or from a legally authorized representative prior to the start of any study-related procedures. This study was conducted in accordance with the Declaration of Helsinki, Good Clinical Practice, International Conference on Harmonization of Technical Requirements for Registration of Pharmaceuticals for Human Use guidelines, EU Clinical Trials Directive, and applicable laws and regulations.

### Study Design

**Figure 1:**
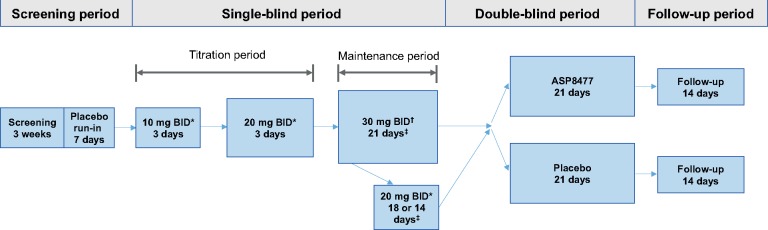
Study design. *Patients who did not tolerate drug dosage were discontinued from the study. ^†^Dose was reduced to 20 mg BID if there were tolerability issues at the 30 mg BID dose. If the reduced dose of 20 mg BID was tolerated, the patient continued on 20 mg BID into the maintenance phase. ^‡^At the end of the maintenance phase, responders to treatments were stratified by disease (PDPN or PHN) and randomized either to continue ASP8477 treatment or to receive placebo. BID = twice daily; PDPN = painful diabetic peripheral neuropathy; PHN = postherpetic neuralgia.

The study consisted of a screening period of up to four weeks (including a one-week, single-blind, placebo run-in period), a single-blind treatment period (approximately four weeks, including a six-day titration period and a three-week maintenance period), a double-blind randomized withdrawal period (three weeks), and a follow-up period (two weeks) (Figure 1). Each patient was assigned a unique identification number at the beginning of the study via interactive response technology (IRT). During screening, patients recorded their daily pain score (via the numeric pain rating scale 0–10 [NPRS]) in an electronic diary (e-diary). After screening assessments, patients who met the inclusion criteria (which included having an average daily pain score ≥ 4) entered a seven-day placebo run-in period. Patients who met daily e-diary compliance criteria (i.e., recorded their daily pain ratings on at least five of seven days, three of which were on the last three days of the week) and had an average pain score between 4 and less than 9 over the last three days of the placebo run-in period were allowed to enter into the single-blind treatment period.

During the single-blind treatment period, patients were initiated on ASP8477 10 mg twice daily (BID) for three days and then escalated to 20 mg BID for a further three days. Patients who tolerated three days of ASP8477 at the 20 mg BID dose entered the three-week single-blind maintenance period at 30 mg BID; patients who did not tolerate either the 10 mg or 20 mg BID dose during the single-blind treatment period were discontinued from the study. During the single-blind maintenance period, patients who did not tolerate ASP8477 after three to seven days of 30 mg BID were permitted to reduce their dose to 20 mg BID and continued treatment on this dose for the remainder of the study. After seven days on the 30 mg BID dose or a reduction to the 20 mg BID dose, no dose modifications were allowed.

Upon completion of the single-blind period, patients who had been compliant with e-diary and were responders to treatment were entered into the three-week, double-blind, randomized withdrawal period. Response to treatment was defined as a 30% or greater decrease in the mean average daily pain intensity during the last three days of the single-blind maintenance period (baseline of the double-blind randomized withdrawal period) compared with the pain intensity at the last three days of the placebo run-in period (baseline of the single-blind period). Eligible patients were stratified based on disease (PDPN or PHN) and randomized in a 1:1 ratio to receive placebo or to continue on the ASP8477 regimen. Patients who did not respond to treatment (nonresponders) and who did not comply with diary entries were discontinued from the study.

To maintain patient blinding during the placebo run-in period and during the single-blind period, patients were unaware of whether they received active ASP8477 or matching placebo tablets. Drug assignment during the placebo run-in, single-blind, and double-blind periods were performed via IRT, and all tablets (ASP8477 and placebo) looked identical. For the three-week, double-blind period, patients were randomized using IRT in a 1:1 ratio to receive placebo or to continue on the ASP8477 regimen.

### Study Subjects

Adult patients (aged ≥ 18 years) with PNP resulting from PDPN or PHN were eligible for enrollment. Eligible patients with PDPN included those with a history of PDPN for one or more years; an established diagnosis of diabetes (type I or II) with PDPN; HbA1c of 11% or lower at screening; stable glycemic control for at least three months prior to screening; and stable diabetic distal symmetrical polyneuropathy symptoms (including pain) for at least three months prior to screening (based on the investigator’s judgment and patient-reported medical history). Eligible patients with PHN were required to have pain present for six or more months after healing of herpes zoster rash. All patients were required to have an average daily pain score of 4 or higher during the screening period, and a mean average daily pain score between 4 and less than 9 out of 10 over the last three days of the seven-day placebo run-in period. All patients were required to maintain their current medications at the same dose throughout the study. Patients taking chronic pain medications were required to be on a stable regimen for one or more months prior to screening. The stable regimen could not include occasional or as-needed (PRN) analgesia or PRN nonmedication therapy. Concomitant medications permitted during the trial included stable medications for neuropathic pain (excluding cannabinoids and opioids), ketoprofen (allowed as rescue medication for up to 12 hours before each visit during placebo run-in and single-blind periods only), and stable medications for chronic diseases (e.g., diabetes, heart disease). No rescue medication was allowed during the double-blind period.

Patients were excluded if they had significant pain from an etiology other than PDPN or PHN that could interfere with assessment of PDPN- or PHN-related pain, a history of (within one year) or current orthostatic (postural) hypotension and associated symptoms, and a history of adverse reactions or clinically significant intolerance to nonsteroidal anti-inflammatory drugs or ASP8477 medication. Patients were also excluded if they had a Hospital Anxiety and Depression Scale (HADS) score higher than 12 on either subscale, an established history of major depressive disorder not controlled with medication, or used opioids for pain for more than four days in the week preceding screening or cannabinoids during the three months prior to screening. Prohibited concomitant medications, therapies, or surgical procedures included strong inhibitors or inducers of cytochrome P-450 enzymes, specifically 2C8, 2C9, or 3A, medications that are almost exclusively metabolized by CYP2C19 and/or CYP2D6 and have a narrow therapeutic window, benzodiazepines, transcutaneous electrical nerve stimulation machines, heat and cold packs, and acupuncture.

### Study End Points

The primary end point was change in mean 24-hour average pain intensity (NPRS) from the double-blind baseline (last three days of the single-blind period) to the last three days of the double-blind randomized withdrawal period. The key secondary end point was time-to-treatment failure during the double-blind period. Treatment failure was defined as the occurrence of three consecutive days during which mean 24-hour NPRS was 4 or higher, and with at least a 30% increase in pain intensity (on each day) relative to baseline of the double-blind period, with the time-to-treatment failure being the first of these three consecutive days [18]. Other secondary end points included responder rate during the single-blind period and safety and tolerability of ASP8477. Exploratory end points included pharmacokinetic (PK) and pharmacodynamic (PD) profiles, Patient Global Impression of Change (PGIC), sleep interference score, European quality of life (EQoL) assessment, HADS score, and neuropathic pain symptom inventory (NPSI).

### Study Assessments

All patient-reported assessments, including all efficacy and sleep interference, were recorded using a handheld e-diary. Quality of life improvements were assessed using the EQ-5D level 5 instrument, which was completed by all patients during visits at the beginning of the single-blind and beginning and end of the double-blind periods. PGIC was assessed by a self-administered seven-grade scale that evaluated patient’s clinical condition relative to the end of the single-blind period. HADS and NPSI were assessed using a self-report scale and a self-report questionnaire, respectively, which were completed by patients during visits at the beginning of the single-blind and end of the double-blind periods. HADS was also assessed at the beginning of the screening period.

Safety and tolerability were assessed throughout the study via adverse event (AE) monitoring (classified according to Medical Dictionary for Regulatory Activities MedDRA v14.0), electrocardiogram measurements, physical examinations, vital sign measurements, and clinical laboratory evaluations. The severity of AEs was classified as mild (no disruption of normal daily activities), moderate (affects normal daily activities), or severe (inability to perform daily activities). Plasma concentrations of ASP8477 at predose and at one, two, four, or six hours postdose were measured in responders and nonresponders at the end of the single-blind maintenance period. Blood samples were also collected for the measurement of the FAAH substrates (AEA, OEA, and PEA) at single-blind baseline, predose, and four hours postdose at the end of the single-blind maintenance period, predose at double-blind baseline, predose, and four hours postdose seven days into the double-blind period, and two weeks after the last dose of study medication.

### Statistical Analyses

A sample size of 30 patients per treatment group in the responder population was estimated to provide an 80% power to detect an effect size of 0.67 (mean treatment difference/pooled standard deviation) at the one-sided significance level of 5%. Data were analyzed based on predefined study populations. Full analysis set 1 (FAS1) included all patients who started the single-blind period and received at least one dose of study drug. Full analysis set 2 (FAS2) included all patients who were responders (≥30% decrease in mean average daily pain intensity over the single-blind treatment phase), received at least one dose of double-blind study drug after randomization, had a pain intensity score at double-blind baseline, and had at least one pain intensity score postbaseline during the double-blind treatment period. Safety analysis set 1 (SAF1) included all patients who took at least one dose of study medication during the placebo run-in period. Safety analysis set 2 (SAF2) included all patients who took at least one dose of double-blind study medication after randomization. PK analysis set included all patients in the FAS1 population for whom at least one quantifiable plasma concentration of ASP8477 was obtained and for whom the time of dosing on the day of sampling was known. PD analysis set 1 included all patients from the FAS1 population for whom sufficient PD measurements were collected. PD analysis set 2 included all patients from the SAF2 population for whom sufficient PD measurements were collected.

All continuous end points and time-to-treatment failure were tested using a one-sided 5% significance level, with corresponding one-sided 95% confidence intervals. The primary end point was analyzed using an analysis of covariance (ANCOVA) model with model terms for treatment group, pooled site, and baseline NPRS score as a covariate. The double-blind mean 24-hour average NPRS scores were analyzed using a repeated measures mixed model with model terms for treatment group, time, time by treatment group, pooled site, baseline NPRS score, and time by baseline NPRS score. The time-to-treatment failure was summarized using Kaplan–Meier estimates and analyzed using the Cox proportional hazards model with model terms for treatment, pooled sites, and baseline NPRS score. Of note, the stratification parameter of disease (PHN or PDPN) was not included in the analysis models due to the very small number of patients included in the double-blind period with PHN. Summaries for the single-blind period were based on FAS1 and included the total number of patients who took ASP8477. Summaries for the double-blind randomized withdrawal period were based on SAF2 for safety analyses and on FAS2 for efficacy analyses.

## Results

### Patient Disposition

**Figure 2:**
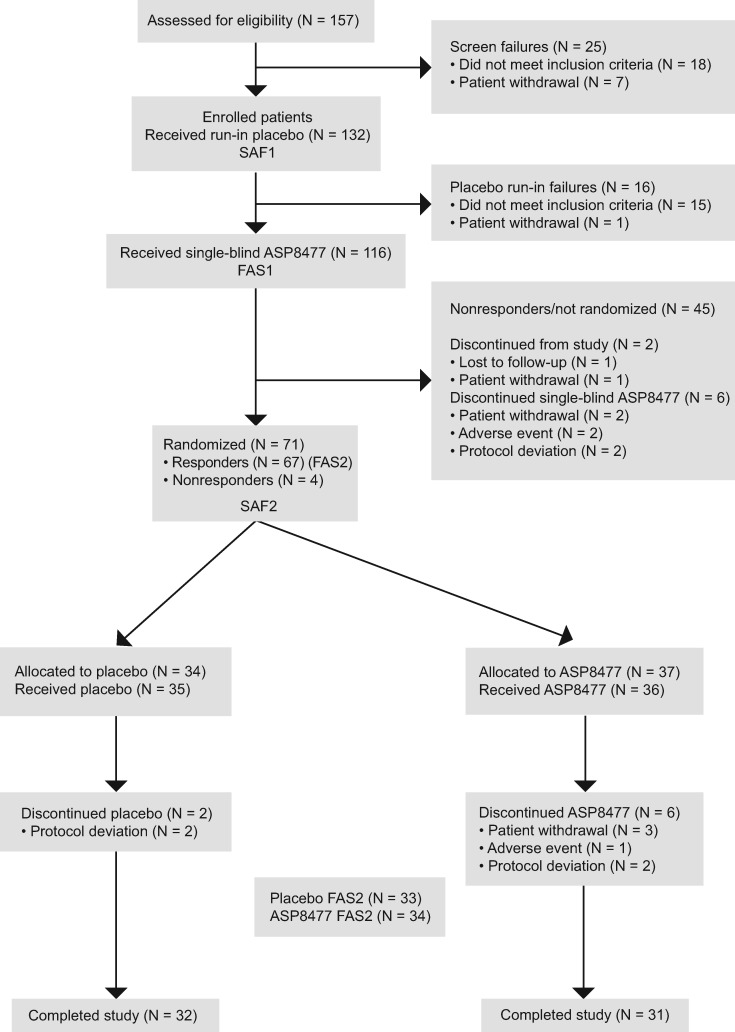
Patient disposition. The four nonresponders who were randomized by mistake were excluded from FAS2 according to a priori definition, resulting in a total of 67 patients in FAS2. One patient in FAS2 who was assigned to the ASP8477 arm received placebo in error. For the purpose of analysis, this patient was therefore assigned to the ASP8477 arm for efficacy analyses on FAS2 and to the placebo arm for safety analyses on SAF2. FAS = full analysis set; SAF = safety analysis set.

A total of 132 patients were enrolled and entered the placebo run-in period (SAF1). During the placebo run-in period, 16 patients discontinued treatment; of these, 15 (11%) did not maintain mean average daily pain scores between 4 and less than 9 out of 10 over the last three days of the placebo run-in period and one (1%) withdrew consent. A total of 116 patients entered the single-blind period and received ASP8477 (FAS1). During the single-blind period, 67 patients (58%) were treatment responders and were randomized to the double-blind period. However, four nonresponders were randomized to the double-blind period in error; hence a total of 71 patients were entered (ASP8477: N = 37; placebo: N = 34; SAF2). One patient who was assigned to the ASP8477 arm received placebo in error; based on intention-to-treat analysis principles, the patient was assigned to the ASP8477 arm (i.e., analysis as randomized) for efficacy analyses on FAS2 and to the placebo arm (i.e., summary as treated) for safety analyses on SAF2. The double-blind randomized withdrawal period was completed by 63 patients (ASP8477: N = 31; placebo: N = 32) (Figure 2).

### Patient Demographics and Baseline Disease Characteristics

Demographic and baseline disease characteristics were similar across all study groups (Table 1). Of the 132 patients enrolled, 131 (99.2%) were Caucasian and one (0.8%) was Asian; approximately 90% of patients had PDPN. During the single-blind maintenance period, three patients had their dose of ASP8477 reduced from 30 mg BID to 20 mg BID. The mean daily dose of ASP8477 during the single-blind maintenance and double-blind periods (ASP8477 arm) was similar (59.8 mg and 58.9 mg, respectively). During both the single- and double-blind periods, all patients received at least one concomitant medication (N = 116 and N = 71, respectively). The most common concomitant medications during the single- and double-blind periods were drugs used in diabetes, agents acting on the renin-angiotensin system, and lipid-modifying agents. The number of patients who took concomitant pain medication in the single-blind period was 65 (56%). Twenty-two patients (59%) and 14 patients (41%) in the ASP8477 and placebo arms, respectively, took concomitant pain medication during the double-blind period.

**Table 1:** Patient demographics and baseline disease characteristics

	SAF1	SAF2	
	(N = 132)	Placebo (N = 34)	ASP8477 10/20/30 mg BID (N = 37*)
Sex, No. (%)			
Male	82 (62.1)	25 (73.5)	17 (45.9)
Female	50 (37.9)	9 (26.5)	20 (54.1)
Age, mean (SD), y	62.7 (9.1)	62.4 (6.6)	62.2 (10.5)
Weight, mean (SD), kg	90.4 (15.9)	89.9 (16.2)	89.1 (17.6)
Medical condition, No. (%)			
Postherpetic neuralgia	12 (9.1)	1 (2.9)	2 (5.4)
Painful diabetic peripheral neuropathy	120 (90.9)	33 (97.1)	35 (94.6)
Time since start of condition, mean (SD), mo	61.9 (51.7)	73.7 (59.1)	67.0 (54.9)

BID = twice daily; SAF = safety analysis set.

* Patients received ASP8477 in both the single-blind and double-blind periods.

### Efficacy

**Figure 3:**
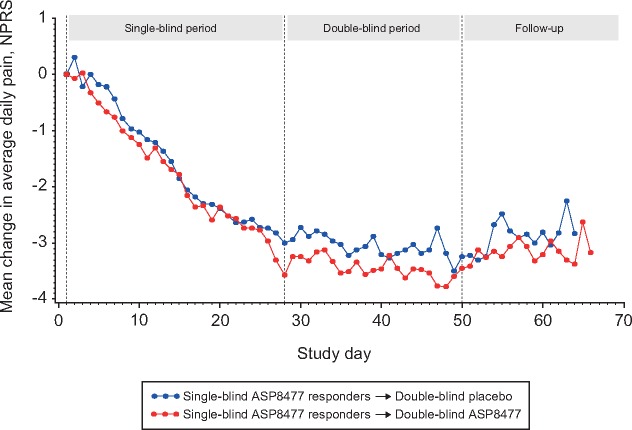
Mean change from single-blind baseline 24-hour NPRS score (FAS2). FAS = full analysis set; NPRS = numeric pain rating scale.

**Figure 4:**
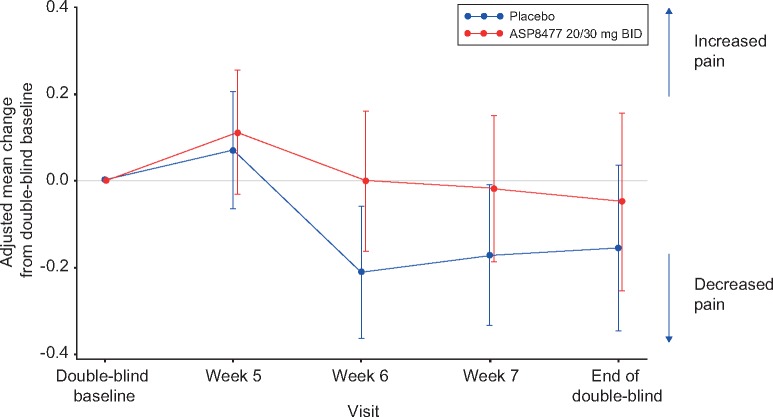
Adjusted mean change from double-blind baseline 24-hour average NPRS score (FAS2). BID = twice daily; FAS = full analysis set; NPRS = numeric pain rating scale.

Analysis of the primary end point, evaluating change from double-blind baseline in 24-hour NPRS score to end of the double-blind period, showed no clinical benefit of ASP8477 treatment over placebo. The mean change from baseline for the two groups was approximately –0.16 for placebo and –0.05 for ASP8477. NPRS score difference in least squares means was +0.11 (95% CI = – to 0.59, *P *=* *0.644) (Table 2 and Figure 3). A secondary analysis using a repeated measures model found nonsignificant differences in pain between the study arms at each week during the double-blind period (Figure 4).

**Table 2:** Change in mean of 24-hour average NPRS score to the end of double-blind period (FAS2)

	Placebo (N = 33)	ASP8477 (20/30 mg BID) (N = 34)
Double-blind baseline NPRS score*		
N	33	34
Mean (SD)	2.57 (1.11)	3.07 (1.36)
End of double-blind period NPRS score^†^		
N	33	33
Mean (SD)	2.45 (1.32)	2.94 (1.61)
Adjusted difference ASP8477—placebo^‡^		
N	33	33
LS mean (SE)		+0.11 (0.29)
One-sided 95% CI		(− to 0.59)
* P*^§^		0.644

CI = confidence interval; FAS = full analysis set; LS = least squares; NPRS = numeric pain rating scale.

* Double-blind baseline NPRS score is defined as the mean 24-hour average pain intensity for the last three days of the single-blind period.

† End of double-blind period NPRS score is defined as the mean 24-hour average pain intensity for the last three days of the double-blind period.

‡ Analysis of covariance model with treatment group and pooled sites as fixed factors and baseline as a covariate.

§ One-sided *P* values shown from analysis of covariance model.

**Figure 5:**
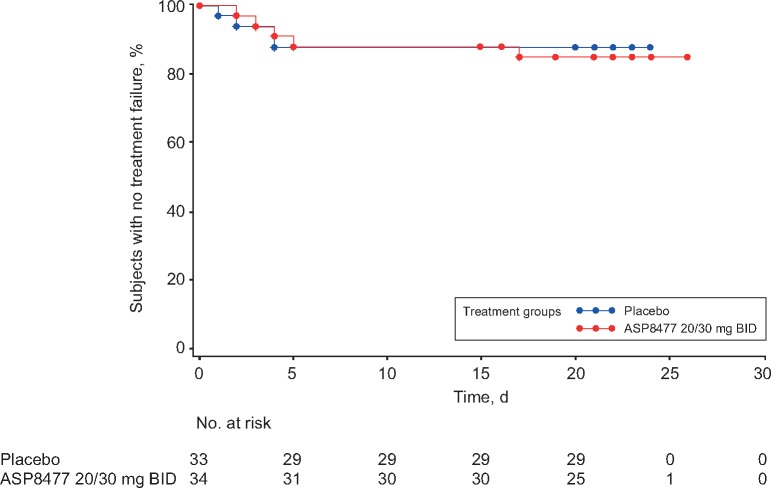
Time-to-treatment failure during the double-blind period (FAS2). BID = twice daily; FAS = full analysis set.

Five patients (15%) in the ASP8477 arm and four patients (12%) in the placebo arm experienced treatment failure in the double-blind randomized withdrawal period (Figure 5). The hazard ratio was 0.97 (95% CI = – to 3.73]), showing no difference in time-to-treatment failure between the placebo and the ASP8477 arms (*P *=* *0.485) (Supplementary Table 1). At the end of the single-blind period, 57.8% of patients were responders. Administration of ASP8477 30 mg BID resulted in an overall mean percent change from single-blind baseline in NPRS score of –35.9% (Supplementary Table 2). No significant difference was observed between ASP8477 and placebo in other exploratory efficacy or profiling measures such as HADS score, PGIC, EQoL assessments, sleep interference, and NPSI score.

### Pharmacokinetic and Pharmacodynamic Profile

**Figure 6:**
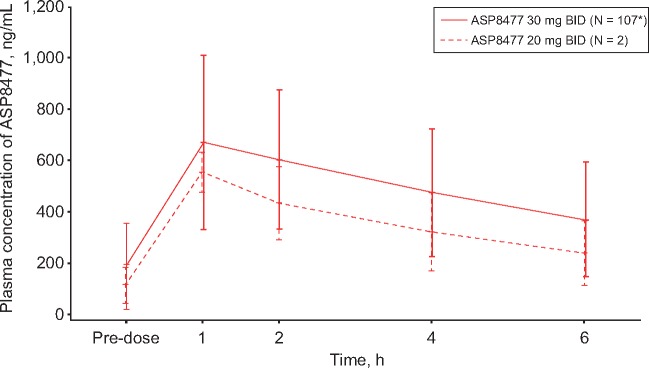
Mean plasma concentration of ASP8477 (PK analysis set). BID = twice daily; PK = pharmacokinetic. *N = 106 at one hour.

At the end of the single-blind maintenance period, mean trough ASP8477 levels were 114 ng/mL (ASP8477 20 mg BID, N = 2) and 190 ng/mL (ASP8477 30 mg BID, N = 107) (Figure 6). Peak plasma concentrations were achieved approximately one hour after taking ASP8477. ASP8477 plasma concentrations gradually decreased thereafter, and by six hours postdose the mean levels were 241 ng/mL (N = 2) in the 20 mg BID and 372 ng/mL (N = 107) in the 30 mg BID dose groups (Figure 6).

The single-blind baseline mean plasma concentrations for the FAAH substrates AEA, OEA, and PEA were 0.45 ng/mL, 2.32 ng/mL, and 2.08 ng/mL, respectively. By the end of the single-blind maintenance period, the levels of AEA, OEA, and PEA more than doubled their baseline levels, with the greatest increases seen in AEA (Supplementary Table 3). At the end of dosing in the double-blind period, the levels returned to baseline values in the placebo group but remained elevated in the ASP8477 groups. AEA, OEA, and PEA returned to baseline values two weeks after the last dose of study drug (Supplementary Table 4).

### Safety/Tolerability

Overall, ASP8477 was well tolerated, with a good safety profile in both the single- and double-blind periods. No deaths were reported during the study. Serious AEs (SAEs) were reported in two patients. During the single-blind period, one patient experienced two serious treatment-emergent AEs (TEAEs), acute renal failure (deemed by the investigator as “moderate severity”) and constipation (deemed as “severe”). Neither was considered related to treatment. During the double-blind period, one patient in the placebo arm experienced two serious TEAEs (acute myocardial infarction and osteomyelitis); both events were judged as severe, but neither was considered related to treatment. Other than the aforementioned SAEs, no other TEAEs were judged to be severe in the ASP8477 arm.

During the single-blind period, 26 patients (22%) experienced at least one TEAE. The most commonly reported TEAE was peripheral edema, experienced by three patients (2.6%) (Table 3), none of whom was on pregabalin or gabapentin prior to or during the study. During the double-blind period, a total of three patients (8%) in the ASP8477 arm and six patients (18%) in the placebo arm experienced at least one TEAE. No individual TEAEs were reported in more than one patient in the double-blind period (Table 4).

**Table 3:** Treatment-related adverse events during the single-blind period occurring in ≥2 patients (FAS1)

Adverse event	ASP8477 20/40/60 mg (N = 116)
Number of patients, No. (%)	26 (22.4)
Peripheral edema	3 (2.6)
Burning sensation	2 (1.7)
Constipation	2 (1.7)
Disorientation	2 (1.7)
Dizziness	2 (1.7)
Myalgia	2 (1.7)
Nasopharyngitis	2 (1.7)
Pruritus	2 (1.7)
Pyrexia	2 (1.7)
Sensation of heaviness	2 (1.7)
Somnolence	2 (1.7)

Only adverse events that started or worsened in the single-blind period were counted as occurring in the single-blind period.

FAS = full analysis set.

**Table 4:** Treatment-related adverse events during the double-blind withdrawal period (SAF2)

Adverse event	Placebo (N = 34)	ASP8477 40/60 mg (N = 37)
Number of patients, No. (%)	6 (17.6)	3 (8.1)
Allergic dermatitis	0	1 (2.7)
Increased appetite	0	1 (2.7)
Musculoskeletal stiffness	0	1 (2.7)
Acute myocardial infarction	1 (2.9)	0
Diabetic foot	1 (2.9)	0
Diarrhea	1 (2.9)	0
Dizziness	1 (2.9)	0
Dyslipidemia	1 (2.9)	0
Dyspepsia	1 (2.9)	0
Hyperuricemia	1 (2.9)	0
Hypoglycemia	1 (2.9)	0
Nasopharyngitis	1 (2.9)	0
Osteomyelitis	1 (2.9)	0

Only TEAEs that started (or worsened) during the double-blind randomized withdrawal period and up to the EoS visit were counted as occurring in the double-blind period. Two patients in the placebo arm and three patients in the ASP8477 arm experienced a treatment-related TEAE.

EoS = end of study; SAF = safety analysis set; TEAE = treatment-emergent adverse event.

Overall, three patients discontinued treatment due to TEAEs. Two patients in the single-blind period experienced two TEAEs of moderate severity (burning sensation and pruritus) that led to treatment discontinuation. The TEAEs in one of these patients were considered to be probably related to the study drug. During the double-blind period, one patient in the ASP8477 arm experienced allergic dermatitis that was considered to be probably related to the study drug.

There were no clinically significant effects on laboratory parameters and no clinically significant liver chemistry abnormalities. During the double-blind period, five mild laboratory-related TEAEs (hypoglycemia, hyperuricemia, and dyslipidemia) were observed in two patients, both in the placebo arm. There was no apparent effect on vital signs or orthostatic blood pressure measures related to study drug administration or dose levels.

## Discussion

This phase IIa EERW MOBILE study evaluated the analgesic efficacy, safety, and tolerability of ASP8477 in patients with PNP resulting from PDPN or PHN. Plasma concentrations of ASP8477 and substrates of FAAH inhibition (AEA, OEA, and PEA) were also evaluated. During the single-blind period, nearly 60% of patients who received ASP8477 met the responder criteria. During the double-blind period, the ASP8477 and placebo groups maintained the levels of improvement observed at the end of the single-blind period. No significant treatment differences in 24-hour average NPRS score or time-to-treatment failure were detected at the end of the double-blind randomized withdrawal period, which was also confirmed by other secondary efficacy analyses. Although one patient, who was randomized to the ASP8477 group, received placebo, the results of the primary analyses were not affected by this; a sensitivity analysis that included this patient in the placebo group (i.e., analyzed as treated) had similar results to the primary efficacy analysis and did not change the conclusions of the withdrawal period. PK/PD analyses suggest that target exposures were reached, resulting in expected increases in targeted peripheral biomarkers (AEA, OEA, and PEA).

Overall, ASP8477 was well tolerated and had a good safety profile in both the single- and double-blind periods. Of note, during the double-blind period, fewer AEs were reported in the ASP8477 arm compared with the placebo arm. This may be due to patients reporting TEAEs that started (or worsened) during the double-blind randomized withdrawal period as occurring in the double-blind period or may be related to the potential withdrawal effects of discontinuing ASP8477 treatment.

Although the present study failed to meet its primary end point, nearly 60% of patients reported a reduction of 30% or more in their pain during the single-blind period, suggesting the potential analgesic activity of ASP8477 in a subset of patients with PNP. However, the lack of worsening pain when patients were switched from ASP8477 (in the single-blind period) to placebo (withdrawal group, in the double-blind period) makes it difficult to interpret the results from the single-blind period but suggests that the single-blind effect may not have been primarily due to the effect of an active treatment. Despite promising preclinical and phase I biomarker results, the clinical evidence supporting FAAH inhibitors for the treatment of pain has not been established in either the current study or other studies that are in phase II development. FAAH inhibition as a treatment for patients with osteoarthritis pain showed no analgesic effect, compared with naproxen, in a phase II proof-of-concept study [19], despite promising findings in preclinical models [20] and healthy volunteers [21]. Additionally, despite elevated endocannabinoid levels in the brains of patients administered the study medication in a phase I study, a FAAH inhibitor as a treatment for neuropathic pain resulting from spinal cord injury failed to meet its primary end point in a phase II proof-of-concept study [22,23].

The limitations in the MOBILE study may have contributed to the lack of treatment effects with ASP8477. In this study, the sample size was small and there was large variability associated with the primary end point. Furthermore, although four countries were selected to participate in this study and three of these enrolled patients, only patients from participating centers in the Czech Republic and Poland ended up being randomized. Therefore, data in this study may not be representative of a wider PNP population. Moreover, study participants were not blinded to the inclusion criteria or to the time of randomization; likewise, investigators were not blinded to entry criteria for either the single-blind or double-blind periods, which may have influenced patients’ perception and reporting of pain. In addition, response to ASP8477 treatment during the single-blind period was not confirmed as stable because patients were required to meet responder criteria only once (i.e., during the last three days of the maintenance period) rather than over a longer period of time during the maintenance period. Verifying stability of ASP8477 treatment over a period of weeks would be more consistent with a maintenance design. The trial design may also be a limitation, as discussed below.

This study highlights the importance of trial design when studying drugs for the treatment of pain, as with many other conditions. The EERW design was used in the MOBILE study because it has been reported to be useful in evaluating drugs that may only benefit a subset of a disease population [24–26]. In addition to proof-of-concept studies in PNP [18,27], EERW has also been used in phase III trials in patients with fibromyalgia [28] and lumbosacral radiculopathy [29]. However, it should be noted that the drugs assessed in these trials had already been shown to be efficacious in pain conditions [18,27–29]. Although the EERW study design is complex compared with conventional or traditional study designs, it may avoid the false conclusion of lack of efficacy and may capture the reality of the range of response to pain therapies often seen in clinical practice [25]. The Initiative on Methods, Measurement, and Pain Assessment in Clinical Trials recommendations highlighted that EERW may provide greater assay sensitivity when randomization is limited to subjects who have demonstrated a clinically meaningful treatment response during the single-blind period [30]; indeed, evidence supporting an increase in assay sensitivity with EERW study design has previously been reported [18,31]. Furthermore, the EERW design allows for the time-to-treatment failure end point that may provide a greater statistical power than mean pain intensity [18]. Although the EERW study has been criticized for limitations in generalizability, where treatment effectiveness is only demonstrated in patients who have already shown response to treatment, it has been argued that this is a benefit of the design as any response shown during the single-blind period cannot be confirmed without the placebo comparison in the double-blind withdrawal phase [32].

However, there are other limitations with the EERW study design; the present study was designed based on a previous report [18]. The overall duration of the present study was short, but the withdrawal period was particularly short especially considering that the duration of the effects of ASP8477 had not been established in phase I studies. As such, there is a potential for a carryover effect of ASP8477 in the present study. Although the phase I data suggest that ASP8477 and endocannabinoid concentrations in the blood are not sustained following the administration of ASP8477 (unpublished data), a prolonged PD effect cannot be ruled out based on these data. A prolonged duration of the double-blind phase in the EERW study design may provide adequate time for a meaningful increase in pain to occur in the placebo group [32]. Furthermore, because EERW trials select only patients who responded to treatments during the single-blind period to continue into the double-blind randomized withdrawal period, awareness of the beginning of the withdrawal period could have influenced patient perception of the effectiveness of the treatment and/or increased the likelihood of placebo effects in responders who were assigned to the placebo arm. This potential limitation may have been avoided if all patients were randomized from the single-blind period into the double-blind randomized withdrawal phase (regardless of response to treatment), with the primary analysis conducted on responders only and the patients blinded to commencement of the randomized withdrawal period, as previously reported [18].

In conclusion, in spite of evidence of a peripheral biomarker demonstrating peripheral FAAH inhibition, ASP8477 did not demonstrate a significant treatment difference compared with placebo in an EERW study in patients with PNP. As the primary end point in the current study and other phase II trials with FAAH inhibitors was not met, future development of compounds targeting FAAH inhibition for chronic pain may need to be re-examined. The use of the EERW study design in clinical trials of pain should take into consideration the duration of the withdrawal period to minimize potential carryover effects, as well as incorporate methods to blind patients to the timing of transition between treatment periods. These changes may reduce bias and placebo response and optimize the potential for detection of a drug signal.

## Supplementary Material

Supplementary Data
