# Supplementary material for: The MOBILE Study—A Phase IIa Enriched Enrollment Randomized Withdrawal Trial to Assess the Analgesic Efficacy and Safety of ASP8477, a Fatty Acid Amide Hydrolase Inhibitor, in Patients with Peripheral Neuropathic Pain
Source: Pain Med. 2017 Apr 5;18(12):2388–400. doi: 10.1093/pm/pnx046 (PMC5939857; doi:10.1093/pm/pnx046)
Supplement: Supplementary Data [file pnx046_supp.zip › Supplementary Table 1.docx]

**Supplementary Table 1. Analysis of time-to-treatment failure (FAS2)**

| **Time-to-Treatment Failure Parameter** | **Placebo**  **(n = 33)** | **ASP8477**  **40/60 mg**  **(n = 34)** |
| --- | --- | --- |
| Treatment failures, n (%) | 4 (12.1) | 5 (14.7) |
| Censored*, n (%) | 29 (87.9) | 29 (85.3) |
| Hazard ratio | | 0.97 |
| One-sided 95% CI | | (–, 3.73) |
| *P*-value^†^ | | 0.485 |

CI, confidence interval; FAS, full analysis set.

*Censored patients are those who did not meet the efficacy failure criteria at Visit 10 (end of treatment/early discontinuation).

^†^One-sided *p-*value shown.
