# Supplementary material for: The MOBILE Study—A Phase IIa Enriched Enrollment Randomized Withdrawal Trial to Assess the Analgesic Efficacy and Safety of ASP8477, a Fatty Acid Amide Hydrolase Inhibitor, in Patients with Peripheral Neuropathic Pain
Source: Pain Med. 2017 Apr 5;18(12):2388–400. doi: 10.1093/pm/pnx046 (PMC5939857; doi:10.1093/pm/pnx046)
Supplement: Supplementary Data [file pnx046_supp.zip › Supplementary Table 2.docx]

**Supplementary Table 2. Responder rate during the single-blind period (FAS1)**

|  | **ASP8477**  **20/40/60 mg**  **(n = 116)** |
| --- | --- |
| Single-blind baseline NPRS score (n = 115) | |
| Mean (SD) | 6.2 (1.1) |
| End of single-blind period NPRS score (n = 116) | |
| Mean (SD) | 4.0 (1.9) |
| Percent change from single-blind baseline (n = 115) | |
| Mean (SD) | –35.9 (28.6) |
| Responder (n = 116) (≥30% reduction in NPRS score) | |
| Yes, n (%) | 67 (57.8) |
| No, n (%) | 49 (42.2) |

FAS, full analysis set; NPRS, Numeric Pain Rating Scale; SD, standard deviation.
