# Supplementary material for: The MOBILE Study—A Phase IIa Enriched Enrollment Randomized Withdrawal Trial to Assess the Analgesic Efficacy and Safety of ASP8477, a Fatty Acid Amide Hydrolase Inhibitor, in Patients with Peripheral Neuropathic Pain
Source: Pain Med. 2017 Apr 5;18(12):2388–400. doi: 10.1093/pm/pnx046 (PMC5939857; doi:10.1093/pm/pnx046)
Supplement: Supplementary Data [file pnx046_supp.zip › Supplementary Table 3.docx]

**Supplementary Table 3. Levels of FAAH substrates during single-blind period (PD analysis set 1)**

| **FAAH Substrate**  **Visit** | **Patients,**  **n** | | | **Absolute Values (ng/mL), Mean (SD)** |
| --- | --- | --- | --- | --- |
| AEA | | | | |
| Single-blind baseline | | 113 | 0.45 (0.18) | |
| Day 14 (predose) | | 109 | 2.72 (0.72) | |
| Day 14 (4 h postdose) | | 110 | 2.96 (0.68) | |
| Day 28 (predose) | | 67 | 2.65 (0.89) | |
| OEA | | | | |
| Single-blind baseline | | 113 | 2.32 (0.87) | |
| Day 14 (predose) | | 109 | 10.79 (2.35) | |
| Day 14 (4 h postdose) | | 110 | 11.50 (2.56) | |
| Day 28 (predose) | | 67 | 10.59 (2.69) | |
| PEA | | | | |
| Single-blind baseline | | 113 | 2.08 (0.61) | |
| Day 14 (predose) | | 109 | 5.88 (1.43) | |
| Day 14 (4 h postdose) | | 110 | 6.09 (1.13) | |
| Day 28 (predose) | | 67 | 5.69 (1.26) | |

AEA, N-arachidonoyl-ethanolamide (anandamide); FAAH, fatty acid amide hydrolase; OEA, oleoylethanolamide; PD, pharmacodynamic; PEA, palmitoylethanolamide; SD standard deviation.
