# Supplementary material for: The MOBILE Study—A Phase IIa Enriched Enrollment Randomized Withdrawal Trial to Assess the Analgesic Efficacy and Safety of ASP8477, a Fatty Acid Amide Hydrolase Inhibitor, in Patients with Peripheral Neuropathic Pain
Source: Pain Med. 2017 Apr 5;18(12):2388–400. doi: 10.1093/pm/pnx046 (PMC5939857; doi:10.1093/pm/pnx046)
Supplement: Supplementary Data [file pnx046_supp.zip › Supplementary Table 4.docx]

**Supplementary Table 4. Levels of FAAH substrates during the double-blind period (PD analysis set 2)**

| **FAAH Substrate**  **Visit** | **Patients, n** | **Absolute Values (ng/mL), Mean (SD)** |
| --- | --- | --- |
| **AEA** | | |
| Placebo | | |
| Double-blind baseline | 32 | 2.57 (0.91) |
| Day 35 (predose) | 32 | 0.53 (0.50) |
| Day 35 (4 h postdose) | 32 | 0.31 (0.10) |
| End of study | 33 | 0.45 (0.16) |
| ASP8477 40/60 mg |  |  |
| Double-blind baseline | 34 | 2.73 (0.89) |
| Day 35 (predose) | 35 | 2.44 (1.17) |
| Day 35 (4 h postdose) | 35 | 3.10 (0.83) |
| End of study | 37 | 0.56 (0.47) |
| **OEA** | | |
| Placebo | | |
| Double-blind baseline | 32 | 10.23 (2.37) |
| Day 35 (predose) | 32 | 2.62 (1.66) |
| Day 35 (4 h postdose) | 32 | 1.78 (0.57) |
| End of study | 33 | 2.33 (0.70) |
| ASP8477 40/60 mg |  |  |
| Double-blind baseline | 34 | 10.91 (2.98) |
| Day 35 (predose) | 35 | 9.89 (3.92) |
| Day 35 (4 h postdose) | 35 | 11.64 (2.68) |
| End of study | 37 | 2.66 (1.50) |
| **PEA** | | |
| Placebo | | |
| Double-blind baseline | 32 | 5.55 (1.04) |
| Day 35 (predose) | 32 | 2.20 (0.84) |
| Day 35 (4 h postdose) | 32 | 1.65 (0.45) |
| End of study | 33 | 2.03 (0.54) |
| ASP8477 40/60 mg |  |  |
| Double-blind baseline | 34 | 5.83 (1.46) |
| Day 35 (predose) | 35 | 5.27 (1.80) |
| Day 35 (4 h postdose) | 35 | 6.06 (1.35) |
| End of study | 37 | 2.17 (0.75) |

AEA, N-arachidonoyl-ethanolamide (anandamide); FAAH, fatty acid amide hydrolase; OEA, oleoylethanolamide; PD, pharmacodynamic; PEA, palmitoylethanolamide; SD, standard deviation.
